# Molecular cloning, polymorphism, and functional activity of the bovine and water buffalo *Mx2* gene promoter region

**DOI:** 10.1186/s40064-016-3729-5

**Published:** 2016-12-22

**Authors:** H. A. E. Babiker, T. Saito, Y. Nakatsu, S. Takasuga, M. Morita, Y. Sugimoto, J. Ueda, T. Watanabe

**Affiliations:** 1 Graduate School of Agriculture, Hokkaido University, Sapporo, 060-8589 Japan; 2 Shirakawa Institute of Animal Genetics, Livestock Technology Association, Shirakawa, Fukushima 961-8061 Japan; 3 Institute of Dairy Science, Rakuno Gakuen University, Ebetsu, Hokkaido 069-8501 Japan; 4 Faculty of Veterinary Medicine, Khartoum University, P.O. Box 32, Shambat, Khartoum Sudan

## Abstract

**Background:**

Bovine *Mx2* gene sequences were already reported, but further information about the gene properties is not yet available. The objective of the current study was to elucidate the structural properties of the bovine *Mx2* gene mainly the promoter region and its possible functional role. If available, such information would help in assessing the functional properties of the gene, which was reported to confer antiviral action against recombinant VSV.

**Results:**

Examinations on the bovine genomic BAC clone—confirmed to contain the *Mx2* gene—revealed 883-bp sequences. A computer scan unequivocally identified a 788-bp promoter region containing a typical TATA box, three ISREs and other promoter-specific motifs. Comparative analysis of nine bovine genomic DNA samples showed 19 nucleotide substitutions suggesting the existence of five different genotypes in the promoter region. The water buffalo *Mx2* promoter region was determined by using primers based on the bovine *Mx2* promoter region disclosing 893-bp, with 56 substitutions, two insertions, 9 and 1 nt at two different sites. A functional analysis of the putative ISRE indicated that ISRE played a synergetic role in the activation of bovine *Mx2* gene transcription.

**Conclusion:**

Bovine and water buffalo *Mx2* promoter region was identified disclosing, the conserved ISRE, located in the proximal end of the promoter region like other members of the antiviral family, suggesting functional activity under interferon stimulation.

## Background

The observation that some strains of laboratory mice escaped experimental influenza virus infection was the starting point for the currently known antiviral Mx protein (Lindenmann et al. 1963; Hug et al. 1988). The protein was classified in the dynamin superfamily, members of which include large GTPases (Staeheli et al. 1993). A common feature of this family is the formation of high-molecular-weight oligomers inside cells (Kochs et al. 2002). Furthermore, conserved properties of Mx proteins include the presence of tripartite GTPase domains in the N terminal region, a dynamin signature, and a GTP effector domain (GED) containing a leucine zipper motif in the C terminal region, which plays a key function in the antiviral activity (Melén et al. 1992). Members of type I interferon family are responsible for the induction of Mx proteins in association with the activation of the natural immune system in infections with single-strand RNA viruses (Pavlovic et al. 1993). Different isoforms of the protein have been detected in a variety of vertebrates (Horisberger and Gunst 1991), and at least one isoform showed antiviral activity. An example of this is the human Mx protein in which human MxA (corresponding to the Mouse Mx1) was found to confer antiviral activity against infection with single-strand RNA virus, while human MxB was devoid of any antiviral action (Frese et al. 1995).

To date, a uniform mechanism of how Mx proteins induce the antiviral action remains unknown, although the localization of the individual proteins inside the cell is thought to play a role (Lee and Vidal 2002).

The existence of more than one isoform of the *Mx* gene in farm animals, including poultry (Bazzigher et al. 1993; Schumacher et al. 1994; Ko et al. 2002), porcine (Morozumi et al. 2001; Asano et al. 2002), ovine (Charleston and Stewart 1993), and bovine (Ellinwood et al. 1998), strength the probability of implementing genetic selection programs to heighten productivity efficiency in the livestock industry. In addition, association of the Mx protein induction with viral infections highlighted the possibility of using the protein as a marker for acute viral infection to monitor a disease state in livestock (Muller-Doblies et al. 2002, 2004).

The notion that the bovine *Mx* locus retained an antiviral allele due to long and intensive selective pressure from single-strand RNA viruses has increased the advantages of using the gene to examine the real significance of the antiviral role of Mx proteins and has encouraged the search for further properties of the gene. Studies of the structural properties showed the presence of the splicing isoform of bovine Mx1 and Mx1B (Kojima et al. 2003) and described the fine structure of the bovine *Mx1* gene, which was found to consist of 15 exons and a promoter region approximately 1 kb upstream of the 5′-flanking region (Gerardin et al. 2004). A functional analysis of the bovine Mx1 protein revealed the gene’s antiviral action against various viral infections including VSV (Baise et al. 2004). It was also seen to be partially effective against rabies (Leroy et al. 2006) but not against Paramyxoviridae (Leroy et al. 2005). Furthermore, variation between the antiviral role of bovine Mx1 and Mx1a was observed using recombinant VSV (Nakatsu et al. 2004).

Bovine *Mx2* gene sequences (accession no. AF355147) have recently been reported, but further information about the gene properties is not yet available. The aim of our study was to learn more about the structural properties of the bovine *Mx2* gene by focusing on the promoter region and its possible functional role. If available, such information would help in assessing the functional properties of the gene, which was described as conferring antiviral action against recombinant VSV (Babiker et al. 2007).

It become clear that the promoter region of type I interferon-induced genes such as mouse *Mx1* and *Mx2*, human *MxA*, bovine *Mx1*, and porcine *Mx1* contain cytokine binding sites and regulatory factors, mainly the interferon stimulation-responsive element ISRE, GAAA elements, and GC-rich boxes. However, the classical TATA box consensus was reported only in mouse *Mx1* and *Mx2* (Hug et al. 1988; Asano et al. 2002; Chang et al. 1991; Gerardin et al. 2004; Thomas et al. 2006). The presence of at least one ISRE is essential to initiate the transcription of interferon proteins. Previous reports indicated that a conserved proximal ISRE has been detected in almost all the promoter regions of every species possessing the gene as a characteristic feature (Reid et al. 1989; Ronni et al. 1998; Asano et al. 2002; Yap et al. 2003; Altmann et al. 2004). It was noticed that the activity of ISRE in human *MxA* was affected by a single nucleotide polymorphism (Hijikata et al. 2001). Moreover, multiple ISRE have also been seen to participate in activation of the promoter region under interferon stimulation (Hijikata et al. 2000).

In this study we have identified the promoter region of bovine and water buffalo *Mx2* genes. We have also demonstrated the variation between the two species, thus providing evidence about the adaptational modifications that took place in the gene. The presence of the conserved ISRE detected in both genes confirms the gene’s relatedness to members of the antiviral Mx protein family induced by type I interferon. Moreover, the presence of the typical TATA box places the gene in the top position among other corresponding genes detected in domestic animals. Expression analysis results suggested that the entire putative ISRE participate synergistically in the activation of the promoter.

## Methods

### Characterization of the BAC clone containing the bovine *Mx2* gene

The bovine *Mx2* gene, was obtained by scanning a bovine genomic bacterial artificial chromosome library (BAC) using a 240-nt α-^35^P-tagged probe generated by a pair of primers, forward primer bMx2 216F (5′-AGAGGACCAGAGGCACTGAG) and reverse primer bMx2 470R (5′-CAAATTCAGCTGATTGAAGTTGT), based upon sequences of putative exon 2 and 3 of bovine Mx2 cDNA (accession no. AF355147). Clones positive to the hybridization were examined for the presence of the *Mx2* gene by sequencing using M13 primers with Big Dye terminator chemistry (Applied Biosystems, Foster City, CA, USA) on an ABI-301 Genetic Analyzer (Applied Biosystems). The BAC clone identified to contain the *Mx2* gene was transformed into *E. coli* cells JM 109 (Promega, Madison, WI, USA) that were cultured overnight at 37 °C in the presence of 25 µg/ml chloramphenicol. The recombinant plasmid DNA was then extracted from these cells following the standard alkaline lysis method, then resuspended in TE (10 mM Tris–HCl, 1 mM EDTA, pH 8.0) and stored at −20 °C, ready for use as stock.

### Identification of the promoter region and transcription start site of the bovine *Mx2* gene

The gene walking technique was followed to identify the predicted promoter region of bovine *Mx2*. The plasmid DNA was sequenced using reverse primer bMx2 25R (5′-AGAGGACCAGAGGCACTGAG) based upon the sequences of bovine *Mx2* cDNA (accession no. AF355147). Sequencing was performed using Big Dye terminator chemistry (Applied Biosystems) with an ABI Prism 310 Genetic Analyzer (Applied Biosystems) in accordance with the manufacturer’s instructions. The sequencing PCR reaction mix used included 100 ng of plasmid DNA as template, 2 µl of ready-mix reagent, and 1 µl 10× buffer. It was then completed to a total volume of 10 µl with autoclaved distilled water. Cycling was done on Thermal Cycler (Perkin-Elmer, Norwalk, CO, USA), with a protocol consisted of an initial denaturing step at 96 °C for 1 min followed by 25 cycles at 96 °C for 30 s, 50 °C for 30 s, and 66 °C for 4 min. The sequencing results identified 500-bp sequences, the computer-assisted scanning of which identified 21-bp similar to those of the putative exon 1 which was followed by an upstream area of 405-bp containing promoter-specific motifs including three ISRE at nt −85, −111, and −134 upstream. Downstream of exon 1 were 74-bp not specific to bovine *Mx2* gene suggesting the 5′ end of intron 1. Two separate sequencing procedures were carried out using a specific reverse primer bMx2 p285R (5′-TGCCGGAGAACCTGTTAGAC) and forward primer bMx2 p444F (5′-GAGGTGCGTACCCTCACACT) designed from the partial sequences of the promoter to identify the final length of the promoter. Parameters were the same as those described in the first sequencing PCR. The final full-length promoter sequences were scanned for the presence of binding sites for cytokines and regulatory factors.

The transcription start site was determined using the GeneRace^®^ cDNA amplification kit (Invitrogen, Carlsbad, CA, USA). Total RNA was extracted using the acid phenol-guanidinium thiocyanate-choloroform (AGPC) procedure (Chomczynski and Sacchi 1987) with Holstein leucocytes cultured in RPMI 1640 medium (Sigma, St Louis MO, USA) for 41 h after stimulation of *Mx* mRNA expression with 1000 units/ml of recombinant human interferon-*α* 2b (IFN; Intron-A, Schering Corp., Kenilworth, NJ, USA) for 12 h. First-strand cDNA was generated from the obtained total RNA using primers specified by the manufacturers. Initial and nested PCRs were then conducted using forward primers GeneRacer 5′-CGACTGGAGCACGAGGACACTGA and GeneRacer 5′ nested 5′-GGACACTGACATGGACTGAAGGAGTA specific to the kit’s synthetic ploynucleotide adaptor, and using the gene-specific reverse primers bMx2 5′-GGGCCCTAGGGAGTGGATGAG and bMx2 559 5′-GGGACCTTCTCCTCATACTT designed according to the manual provided by the manufacturer to amplify the bovine *Mx2* cDNA. The obtained amplicons were electrophoresed in 1% agarose gel for confirmation, and bands corresponding to the expected cDNA fragments were excised for purification. Purified cDNA fragments were T/A ligated to pGEM vector (Promega) and sequenced to determine the transcription start point of the bovine *Mx2* gene.

### Amplification of the *Mx2* promoter region using bovine and water buffalo genomic DNA

A genomic DNA library derived from one water buffalo and nine bovine blood samples were used to identify the promoter region of the water buffalo *Mx2* gene and to demonstrate the allelic polymorphisms of the bovine *Mx2* promoter region, respectively. The *Mx2* promoter region was amplified from the genomic DNA by PCR using forward primer bMx2 pro788F 5′-AATTTGCCCACAAGTCAGG and reverse primer bMx2 95R 5′-AGACTCGAGAGCCACGTTTATCAGGAAGC designed based upon the promoter sequences, partial 5′UTR, and intron 1, which was identified using the BAC clone, as previously described. The amplicons were electrophoresed in 1% agarose for purification of the target cDNA. The obtained PCR product was then T/A cloned into pGEM easy vector (Promega), and the recombinant plasmid was incorporated into *E. coli* JM 109 (Promega) competent cells. The purified recombinant plasmid was sequenced using promoter-specific primers on an ABI gene analyzer as described before. The sequencing results obtained were subjected to a computer-assisted comparative analysis to examine the promoter-specific motifs and polymorphic properties of the bovine and water buffalo *Mx2* promoter region, respectively, using the website-based tools.

### Characterization of the role of ISRE in promoter region activity

The role of the putative ISRE was examined using the dual-lucifersae reporter system (Promega), which contains promoter-less pGL3 vector expressing the firefly luciferase and internal control plasmid pRL-TK, expressing *Renilla* luciferase. The test was conducted using two types of pGL3 constructs—deletion- and truncation-generated constructs—in accordance with the template used. The deletion-generated constructs included pGL3-(−422), a fragment that include the proximal interferon-sensitive regulatory factors: pGL-(−154), containing the entire ISRE as wild type; pGL-(-132) with the distal ISRE deleted; pGL-(−109), that the distal and intermediate ISRE deleted; and pGL-(−76), where the entire three ISRE deleted. These constructs were established by the T/A cloning of fragments generated from the genomic DNA by progressive deletion relative to the transcription start point using primers (Table 1) for the pGL3 vectors. Truncation-generated constructs included pGL-ΔISRE1 + 2, pGL-ΔISRE1, and pGL-ΔISRE2 which was generated from the pGL-(−154) construct as a template using primers without restriction sites (Table 1). Forward primers used included the *Kpn*I restriction site in the 5′ end, while reverse primers included the *Xho*I restriction site in the 5′ end. The PCR mix reaction included 100 ng template, 10 pmol primer, 75 mM MgSO_4_, 5 µl 10× buffer, 10 mM dNTP mix, and 5 units *Taq* polymerase. The reaction was completed to 24 µl with autoclaved distilled water (DW). The cycling protocol started with initial denaturing at 95 °C for 3 min, followed by 35 cycles of denaturing at 95 °C for 30 s, annealing at 58 °C for 30 s, and extension at 72 °C for 1 min. The PCR product of fragments generated by progressive deletion was checked by agarose gel and purified. The 5′ and 3′ ends were excised with *Kpn*I and *Xho*I, respectively, and ligated to the equivalent sites in pGL3. The recombinant plasmid was transformed into *E. coli* JM 109 (Promega), miniprips extracts of which were checked using RV primer3 5′-CTAGCAAAATAGGCTGTCCC or GL primer2 5′-CTTTATGTTTTTGGCGTCTTCC provided by the manufacturer. The plasmid was purified using the Wizard^®^ PureFection Plasmid DNA Purification System (Promega), TE resuspended, and then the concentration was determined using an Eppendorf^®^ BioPhotometer (Eppendorf AG, Germany). MDBK cells were cultured in DMEM supplemented with 10% FBS, 100 U/ml ampicillin and 100 U/ml streptomycin then transfected in duplicates using FuGENE 6 (Roche Diagnostics, Indianapolis, IN. USA). The MDBK cells were only transfected with pGL3 basic vector as a negative control or with the different pGL3 constructed, as described before simultaneously with pRL-TK constructs as internal control. Briefly, MDBK cells were subcultured in 24-well plates in a total cell count of 2.5 × 10^4^ cell/well and incubated for 24 h for 80–90% confluence. A transfection mixture including 100 µl of serum-free medium, 1.8 µl of FuGENE 6 (Roche), a total concentration 0.5 µg pGL3 constructs, and 0.1 µg internal control pRL-TK (Promega) with a ratio of 5:1, test construct:internal control was incorporated into the cell culture by dropping to secure equal distribution. The cell cultures were incubated for 41 h post transfection in a CO_2_ incubator at 37 °C. Recombinant human interferon-*α* 2b at a concentration of 1000 units/ml was then added into the wells of one set of the duplicates, and the incubation continued for 48 h for both treated and non-treated cells. At the end of the incubation time, the cell cultures were removed from the incubator, washed with PBS, and subjected to lysis with 100 µl of 1× passive lysing buffer. The plate was then rocked on a shaker for 15 min and lysates were removed to 0.5 ml micro tubes, kept on ice for 1 min, and centrifuged for 2 min at 4 °C at maximum speed (15,000 rpm). Finally, 20 µl of the lysates was added to a 96-well plate to run the luciferase assay.

**Table 1:** Primers and templates used in generation of deletion and truncated mutants for luciferase activity test

Construct	Primer name		Primer type	Template
pGL3-(−422)	bMx2 p 422 + *Kpn*I	5′-AGAGGTACCCCGTGGAGGAGCTGTGGATGTC	Forward	Genomic DNA
	bMx2 p 20 + *Xho*I	5′-AGACTCGAGCCTGTGCACTTGTAGGAGGG	Reverse	
pGL-(−154)	bMx2 p154 + *Kpn*I	5′-AGAGGTACCCGCTTCAGGTTTCATTTCTG	Forward	Genomic DNA
	bMx2 p 20 + *Xho*I	5′-AGACTCGAGCCTGTGCACTTGTAGGAGGG	Reverse	
pGL-(−132)	bMx2 p132 *Kpn*I	5′-AGAGGTACCGCAGGCTAATGGTTTCGTTT	Forward	Genomic DNA
	bMx2 p 20 + *Xho*I	5′-AGACTCGAGCCTGTGCACTTGTAGGAGGG	Reverse	
pGL-(−109)	bMx2 p109 + *Kpn*I	5′-AGAGGTACCGGGCAAGCCATTAGTTTCAT	Forward	Genomic DNA
	bMx2 p 20 + *Xho*I	5′-AGACTCGAGCCTGTGCACTTGTAGGAGGG	Reverse	
pGL-(−76)	bMx2 p76 + *Kpn*I	5′-AGAGGTACCGGGAAAGCAAGCCACGAG	Forward	Genomic DNA
	bMx2 p 20 + *Xho*I	5′-AGACTCGAGCCTGTGCACTTGTAGGAGGG	Reverse	
pGL-ΔISRE1 + 2	bMx2 p84F	5′-TTACTTCTGGGAAAGCAAGC	Forward	pGL-(−154)
	bMx2 p127	5′-GCCTGCCACAGAAATGAAAC	Reverse	
pGL-ΔISRE1	bMx2 p84F	5′-TTACTTCTGGGAAAGCAAGC	Forward	pGL-(−154)
	bMx2 p99	5′-ATGGCTTGCCCTAGAAACG	Reverse	
pGL-ΔISRE2	bMx2 p109	5′-GGGCAAGCCATTAGTTTCAT	Forward	pGL-(−154)
	bMx2 p127	5′-GCCTGCCACAGAAATGAAAC	Reverse	

The test was carried out in an automated LD 400C Luminescence detector (Beckman Coulter, CA, USA). A volume of 100 µl Luciferase assay reagent II (Promega) was added to each well and the luciferase activity was measured for 10 s after a 3 s delay. Then 100 µl of Stop&Glo Solution was added to the wells to quench the luminescence of the test constructs and to activate the luminescence of the internal control constructs. The cumulative light emitted was expressed as relative light units (RLU).

### Comparative and computer assisted analysis and biometrics

The similarity between sequences and allelic polymorphisms was investigated using the BLAST programs at http://www.ncbi.nlm.nih.gov/blast/bl2seq/wblast2.cgi/ (Altschul et al. 1990), while the MOTIF program available at http://motif.genome.jp/ was used to search for a promoter-specific motif.

The data obtained from the luciferase activity test was analyzed using STATVIEW software (Abacus Concepts Inc. CA, USA). The statistical significance was analyzed using Fisher’s protected least significant difference test. All the results were expressed as mean values ± standard errors of the mean (SE).

## Results

### Promoter region and transcription start point of the bovine *Mx2* gene

**Fig. 1:**
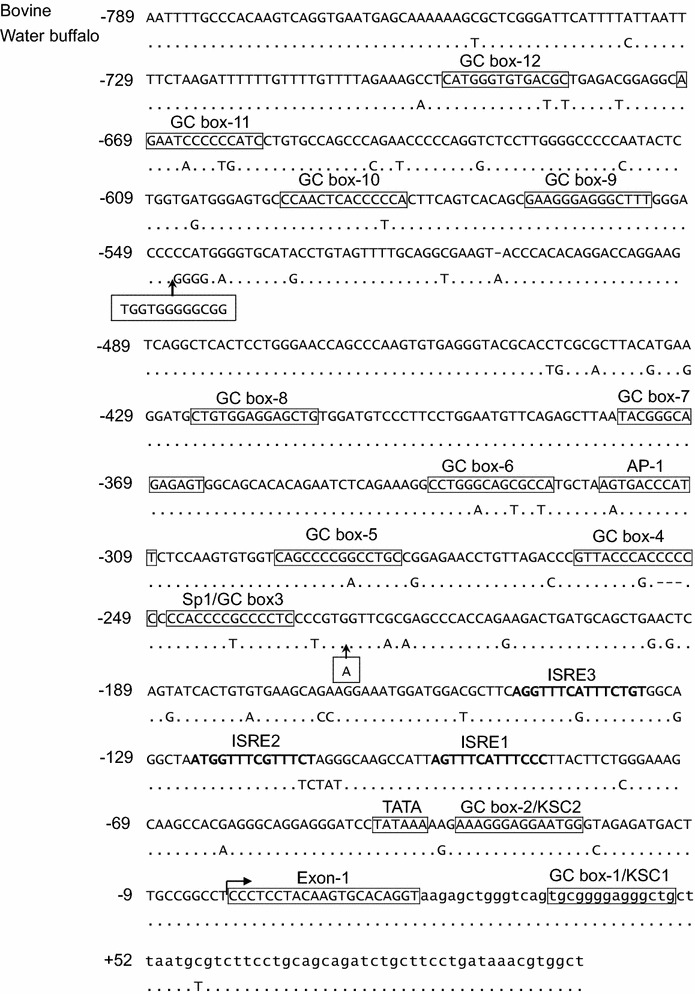
Sequences of bovine *Mx2 gene* promoter region Compared to those of water buffalo *Mx2 gene* promoter region (accession number will be provided after final acceptance) displaying nucleotide substitutions encountered. *Dot symbol* means same as the bovine’s one. *Boxes* indicate the putative transcription and regulatory. *Dashed boxes with arrows* indicate the nucleotide sequences and the position of insertions in water buffalo *Mx2* gene promoter region. *Letters in small cases* indicates sequences of intron 1

The results of the first sequence using the plasmid DNA and reverse primer designed from the putative exon 1 of bovine *Mx2* cDNA sequences (accession no. AF355147) disclosed a 500-bp amplicon. Computer-assisted scanning of the amplicon identified a 21-bp sequence typical to those of the putative exon 1 of bovine *Mx2* cDNA (accession no. AF355147). Moreover, exon 1 was followed by an upstream area of 405-bp including a typical TATA box at nt −39 upstream of the putative transcription start and other promoter specific motifs characterizing the promoter of type I interferon-induced antiviral Mx genes, unequivocally identifying the predicted promoter region. Downstream of exon 1 were 74-bp, expected to belong to intron 1. The result of the second sequencing using primers designed based on the partial sequences of the identified promoter region added about 383 nt. This brought the promoter sequences to 788-bp upstream of the putative transcription start site of the bovine *Mx2* gene. As shown in Fig. 1, computer-assisted scanning of the obtained sequences revealed a typical TATA box at nt −39 upstream, three ISRE at nt −85, −111, and −134 upstream, AP-1 at nt −310 upstream, and 11 GC-rich boxes at nt −22, −234, −249, −282, −325, −364, −411, −553, −580, −656, and −683 upstream of the putative transcription start site. One of these GC rich boxes was found at nt +35 downstream of the 5′ end of intron 1.

The transcription start site was determined by sequencing a total RNA-derived cDNA fragment including a synthetic polynucleotide adaptor in the 5′ end. Observation of the nucleotide sequences obtained identified the nucleotide “C” directly next to the last adaptor-specific nucleotide. Furthermore, computer-assisted scanning of the nucleotide sequences obtained identified exon 1 compared with the promoter and partial 5′UTR sequences obtained using the BAC clone. Therefore, as indicated in Fig. 1, nucleotide “C” was identified as the bovine *Mx2* gene transcription start site.

### Bovine *Mx2* promoter region polymorphism

**Fig. 2:**
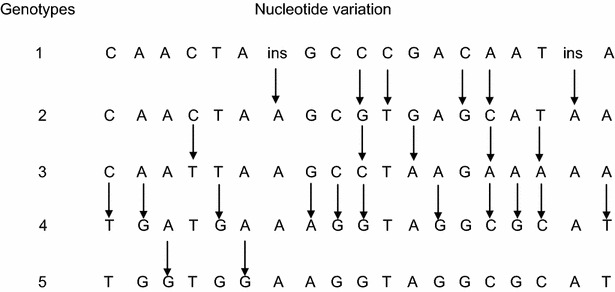
A diagram displaying nucleotide substitutions of the genotypes detected at the promoter region of bovine *Mx2 gene* from the breeds examined. *Arrows* indicate the genetic variations

Sequence analysis of the nine samples of genomic DNA from five bovine breeds using primers based on the promoter region identified in this study revealed an amplicon of 883-bp. Further comparative analysis revealed that these amplicons were highly similar (99%) to the bovine *Mx2* promoter identified using the BAC clone. Moreover, several nucleotide substitutions were detected among the breeds examined with a total number of 19 different nucleotide substitutions. Seventeen of these nucleotide substitutions were located in the promoter and two were detected in intron 1 (Table 2). Similarly, four of these nucleotide substitutions were found in all the samples examined: insertion of adenine at position −509, cytosine to thymine at −230, cytosine to guanine at −190, and insertion of adenine at 23 (Table 2). According to the nucleotide substitution detected, the genomic DNA samples examined were classified into five genotypes (Fig. 2). However, none of these nucleotide substitutions were detected in the putative ISRE or in the exon 1 sequences.

**Table 2:** Nucleotide substitution detected on the promoter region and intron 1 of bovine Mx2 gene

Animals	−742	−704	−614	−582	−542	−532	509	−265	−261	−255	−253	−222	−209	−169	−100	−20	23	53	Alleles
BAC clone	C	A	A	C	T	A	–	A	G	C	C	G	A	A	A	T	–	A	1
JaBla, Brah 2-1,3	–	–	–	C	–	–	A	–	–	–	G	–	–	C	–	–	A	–	2
Charolait	–	–	–	T	–	–	A	–	–	–	–	A	–	–	–	A	A	–	3
Hol 1, 2, 3, Here, Brah 2-2	T	G	–	T	G	–	A	C	A	G	G	A	G	C	G	C	A	T	4
Brah 1	T	G	G	T	G	G	A	C	A	G	G	A	G	C	G	C	A	T	5

(–) symbol means same as the BAC clone’s one

*Ja black* Japanese Black, *Hol* Holstein, *Brah* Brahman

### Promoter region of the water buffalo *Mx2* gene

The sequence result of the amplicon generated from water buffalo genomic DNA using primers designed based upon the bovine *Mx2* promoter region revealed 893-bp to be highly homologous (93%) to the bovine sequences. Computer-assisted scanning of the obtained sequences clearly identified the presence of the TATA box and other promoter-specific motifs that characterize the promoter region of type I interferon-induced genes. These genes include the antiviral *Mx* gene, confirming the water buffalo *Mx2* gene promoter region. Moreover, as shown in Table 2, the scanning result also identified 56 nucleotide substitutions compared with the bovine *Mx2* promoter region, in addition to a 9 nt insertion at position −546 and a 1 nt insertion at position −227. None of these substitutions were detected in the putative ISRE.

### Role of the putative ISRE in the activation of the promoter

**Fig. 3:**
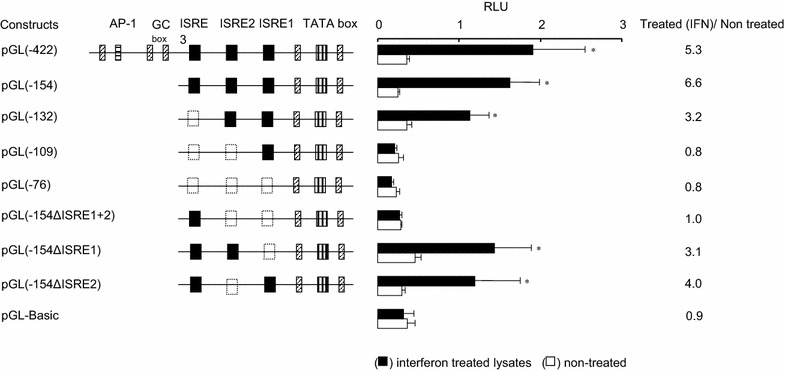
A histogram of activity examinations of the putative ISREs detected in the bovine *Mx2* gene promoter region (*right*), with schematic diagram depicting the putative regulatory factors of each used construct correspondingly (*left*). The luciferae activity test was performed using lysates of interferon treated (*black square*) or non-treated (*white square*) MDBK cell transfected with pGL3 constructs including pGL3-(−422), pGL3-(−154), pGL3-(−132) pGL3-(−109), pGL3-(−76), pGL(−154ΔISRE1 + 2), pGL(−154ΔISRE1), pGL(−154ΔISRE2), and pGL3-basic were transfected simultaneously with pRL-TK. Twenty ml of each lysate was used to measure the luciferase activity on an automated LD 400C Lumicescence detector using the specified reagents and the results are expressed as mean values ± standard errors of the mean (SE)

The role of the putative ISRE was determined by measuring the luciferase activity using lysates of interferon-treated or non-treated MDBK cells transfected with the different pGL3 constructs individually. As shown in Fig. 3, the highest expression level was detected in interferon-treated cells transfected with pGL(−154) construct, which was 6.6-fold higher than the non-treated cells transfected with the same construct. The expression was suppressed dramatically when all or two of the ISRE were deleted [pGL(−154ΔISRE1 + 2), pGL(−109), pGL(−76), with RLU 1.0, 0.8, and 0.8, respectively]. A relative drop in the expression level was detected when only one ISRE was deleted [pGL(−154ΔISRE2), pGL(−132), pGL(−154ΔISRE1) with RLU 4.0, 3.2, and 3.1 respectively]. The negative control pGL3basic transfected/treated or non-treated and non-treated/transfected cells were devoid of significant luciferase activity.

## Discussion

**Fig. 4:**
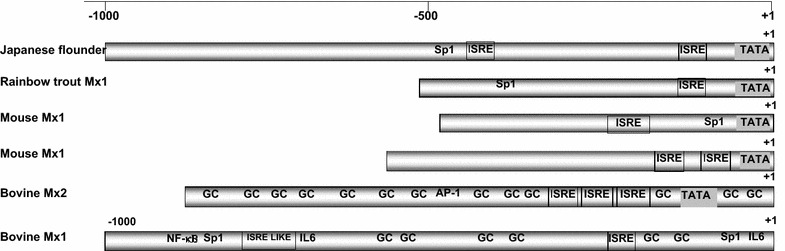
Schematic diagram displaying the variation in the fine structure of promoter regions of bovine *Mx2* (accession number will be provided after final acceptance), Japanese flounder fish (Ooi et al. 2006), rainbow trout *Mx1 (*Trobridge and Leong 1995), mouse *Mx1* (Hug et al. 1988), mouse *Mx2* (Chang et al. 1991), and bovine *Mx1* (Kojima et al. 2003). The transcription start site is indicated by +1. *ISRE* interferon stimulation responsive element, *GC* GC-rich box, *Sp1* specificity protein 1(transcription factor), *AP-1* activator protein 1, *NF-κb* nuclear factor kappa-light-chain-enhancer of activated B cells

The promoter region of type I interferon-induced proteins, including the Mx protein, were demonstrated to contain ISRE, GAAA, and GC-rich box elements related to promoter activity under interferon stimulation in many species (Gerardin et al. 2004). Consistent with this findings, our present analysis of the bovine *Mx2* gene promoter region revealed 788-bp complete sequences of the promoter region with a typical TATA box and three ISRE with several GC-rich boxes and GAAA series characterizing the promoter of type I interferon-induced antiviral *Mx* genes (Fig. 1). This is in accordance with results obtained in a functional analysis of bovine *Mx2* cDNA, which was found to confer antiviral activity against recombinant VSV as a new member of the antiviral Mx protein family (Babiker et al. 2007). The presence of a TATA box was reported in the promoter region of both mouse *Mx1* (Hug et al. 1988) and *Mx2* (Asano et al. 2002), rainbow trout *Mx1* (Trobridge and Leong 1995) and in the Japanese flounder *Mx* gene (Ooi et al. 2006) (Fig. 4), though no functional role was detected for these elements concerning promoter region activity in relation to stimulation with interferon. The detected TATA box placed the bovine Mx2 protein promoter region at the top among the promoter regions of Mx proteins of other mammals, such as human MxA (Chang et al. 1991), bovine Mx1 and porcine Mx1; all of which were reported to lack a typical TATA box (Gerardin et al. 2004; Thomas et al. 2006). However, the presence of three proximal ISRE puts the bovine promoter region in the same group with the human *MxA* and porcine *Mx1* promoter region, apart from the “standard” *MX* promoter of some fish, chicken, and bovine *Mx1*, as claimed by Thomas et al. (2006) (Fig. 4). The sequences of the three ISRE detected in the bovine *Mx2* promoter region currently was in accordance with the conserved sequences found in promoter regions of other species such as bovine *Mx1* and porcine *Mx1* 1(GAAA1-2GAAA(G/C)). No ISRE was found in the intron of the bovine *Mx2* gene. GC-rich boxes are the binding site for Sp1 [a ubiquitous transcription factor in mammals (Husmann et al. 2000)]. The presence of GC-rich boxes in the promoter regions of Mx proteins lacking a TATA box, such as bovine Mx1 and porcine Mx1, was thought to replace the TATA box functionally leading to the transcription suggesting multiple transcription start sites (Gerardin et al. 2004; Thomas et al. 2006). Interestingly, an activator protein element was found in the promoter region of bovine *Mx2*, which is a member of influenza virus-activated cytokine binding sites (Julkunen et al. 2001). Another member of this family is NFκ-B; was detected in the bovine *Mx1* gene promoter region (Gerardin et al. 2004) and found to be more sensitive to activation by the virus (Zamanian-Daryoush et al. 2000).

Results obtained comparing the bovine *Mx2* gene promoter region in nine animals representing five different breeds with those of the BAC clone demonstrated the promoter region’s polymorphism with 19 nucleotide substitutions (Table 2). Accordingly, the bovine *Mx2* promoter region is expected to host one of five different genotypes, as suggested according to these nucleotide substitutions (Fig. 2). However, absence of nucleotide substitution in exon 1 was consistent with a previous study on the polymorphic properties of bovine *Mx2* cDNA (Babiker et al. 2007) as well as bovine *Mx1* (Nakatsu et al. 2004). In addition, although the number of nucleotide substitutions is relatively large compared to those detected in the gene’s cDNA, the gene conservation was evident, that is due to the absence of nucleotide substitution in the transcription regulatory elements characterizing the promoter of the antiviral Mx proteins such as ISRE. The same result was obtained in analysis conducted using the bovine Mx1 protein promoter region (Yamada et al. 2009).

Sequence results of PCR products from water buffalo genomic DNA using primers designed from the sequences of bovine *Mx2* promoter disclosed a homologous (93%) promoter region, an analysis of which revealed the presence of 56 nucleotide substitutions in addition to two inserts at two dependent sites (Table 2). The same result was found in previous studies when cDNA sequences of water buffalo *Mx1* and *Mx2* were compared to bovine *Mx1* and *Mx2* cDNA sequences, respectively (Nakatsu et al. 2004; Babiker et al. 2007). Based upon the bovine *Mx1* gene, these findings substantiate what had already been demonstrated that the Mx protein was a target for adaptational changes (Gerardin et al. 2004).

Previous reports indicated that a proximal ISRE is essential for *Mx* promoter region activity and that ISRE detected in the distal area are non-functional if on their own and may provide support to the proximal ISRE if several are present at the same time (Ronni et al. 1998; Asano et al. 2002; Yap et al. 2003; Altmann et al. 2004). No distal ISRE or ISRE-like elements were detected in the bovine *Mx2* gene promoter region. Therefore, fragments cloned in the pGL3 constructs used in the expression analysis were generated to contain the putative elements found in the proximal end of the promoter region (Fig. 3). The selection of these fragments was also based upon observation driven from similar analysis conducted previously using the bovine *Mx1* promoter region, which suggested that those distal transcription regulatory elements, if available, are almost nonfunctional (Yamada et al. 2009). The results of a luciferase activity test conducted in this study clearly agreed with these findings (Fig. 3). The expression level in constructs containing the three ISRE was the highest among the expression levels obtained using other constructs, even those including all the ISRE, with other regulatory factors near the distal end of the promoter region. It was obvious that the three ISRE participate synergistically in the activation of the promoter region under interferon stimulation. Moreover, in accordance with previous reports, the constructs containing the distal and the proximal ISRE—but not the intermediate—gave 60% activity compared to other combinations.

## Conclusion

In conclusion, we have identified the bovine and water buffalo *Mx2* promoter region. Although conservativeness of the genes was demonstrated, based upon results obtained by observations of promoter regions of the animals and breeds examined, we assume that adaptational changes took place between the species. Moreover, like other members of the antiviral family, the bovine *Mx2* promoter region disclosed the conserved ISRE, located in the proximal end of the promoter region, suggesting functional activity under interferon stimulation.

## Abbreviations

AGPC: acid guanidinium phenol chloroform
cDNA: complementary deoxyribonucleic acid
DMEM: Dulbecco’s modified Eagle’s medium
DNA: deoxyribonucleic acid
dNTP: deoxyribonucleotide triphosphate
FBS: fetal bovine serum
GTPase: guanosine 5′-triphosphatase
IFN: interferon
ISRE: interferon stimulated response sequence
PCR: polymerase chain reaction
MDBK: Madin–Darby bovine kidney (MDBK) cells
mRNA: messenger ribonucleic acid
RNA: ribonucleic acid
RT: reverse transcription
TE: tris ethylenediamine tetraacetic acid
VSV: vesicular stomatitis virus
